# Comparison of Epoxy Resin and Rigid Polyurethane Foam as a Bone Substitute to Study Implant Biomechanics

**DOI:** 10.7759/cureus.79124

**Published:** 2025-02-16

**Authors:** Nida Zehra Bano, Aqsa Shaukat

**Affiliations:** 1 Department of Science of Dental Materials, Army Medical College, National University of Medical Sciences, Rawalpindi, PAK

**Keywords:** biomechanics, bone substitutes, dental implants, dental stress analysis, implant-supported prosthesis, polyurethane foam

## Abstract

Introduction

The study of biomechanical factors is crucial for discerning the solutions relating to implant failures. These factors are analyzed using in vitro models. These models help us to understand the relation of these variables with implant biomechanics. Multiple materials are available for use as bone substitutes for in vitro studies. Each material has its own pros and cons, and there is no single material that can ideally replicate bone behavior under intra-oral conditions.

Objectives

The objective of this study was to synthesize and compare the two synthetic bone substitute materials as a biomechanical bone model to evaluate the stress distribution in the peri-implant region using strain gauge analysis.

Materials and methods

The two bone substitutes used were polyurethane (PU) foam (Sawbones, Vashon, Washington, United States) and epoxy resin (Polycraft ClearTop 35 Epoxy Water Clear Resin System, MB Fibreglass, Newtownabbey, Northern Ireland). The PU models were designed in two densities to mimic bone type 2 as per the Misch classification. A mold was used for the epoxy resin models. Dental implants of two different thread designs were used for biomechanical load transfer. The implants were placed in both types of models using the manufacturer's instructions. A strain gauge was bonded over each model adjacent to the upper third (coronal) region of each implant. The strain gauge was connected to the strain meter. A combined (axial and non-axial) loading ranging from 50 N to 300 N magnitude was applied using a universal testing machine (UTM). The load was applied using a porcelain fused to a metal prosthesis bonded over a bolt head which was fixed in the crosshead of the UTM. The strain rate was 0.95 mm/min, and the strain values were recorded using a strain meter. The collected data was organized and analyzed using IBM SPSS Statistics for Windows, Version 22.0 (Released 2013; IBM Corp., Armonk, New York, United States).

Results

All strain values recorded were less than 3000 µε and within the physiological loading zone per the Frost theory. The difference between the strains produced in both models was statistically significant (p≤0.05). The strain values recorded were higher in the PU model as compared to epoxy resin.

Conclusions

PU foam can be preferred as a bone substitute in biomechanical studies as it provides a more accurate estimation of strain in the peri-implant region. Its wide range of available densities can allow researchers to mimic various types of bones, facilitating the analysis of stress and strain distribution produced by the implants under study.

## Introduction

Bone is the structural foundation for a load-carrying implant [1]. The density of available bone at the surgical site determines treatment planning and prognosis [2]. The bone density varies with location. The mandible is an independent bone, acting as a force-absorbing unit as compared to the maxilla which acts as a force-distributing unit. Due to this reason, the bone in the maxilla is less dense [2].

Biomechanical overloading leads to the late failure of an implant occurring after the placement of a prosthesis [1]. Overloading produces excessive strain in the bone resulting in marginal bone loss and eventual implant failure. Many studies have figured that the biomechanical factors affect the prognosis more as compared to the biological factors and they must be considered for long-term success [3,4]. So, the study of biomechanical factors is crucial for extrapolating the solutions relating to implant failures.

Implant failure can be studied using both clinical and biomechanical research, with clinical studies being considered more practical and applicable than biomechanical tests. However, these studies have limitations of inherent variability due to multiple uncontrolled factors such as patient compliance, oral hygiene conditions, and occlusal/masticatory profiles. Compared with clinical studies, biomechanical studies may provide several powerful advantages.

Biomechanical investigations are generally more cost-effective and cost-efficient compared to clinical studies. It is low in expenses and time requirements by a factor of 10 [5]. The testing environments can be meticulously controlled and repeatable. Specific quantitative parameters are used to derive results, as opposed to clinical research which often utilizes indirect assessments and outcome measures. In vitro biomechanical studies are often more sensitive in detecting differences between groups compared to clinical studies [5]. 

Biomechanical research can be very influential and valuable in bone-related fields. Data from a single study if carefully curated and analyzed can provide us valuable information that otherwise can take years to deduce. For example, in the field of hip arthroplasty, it took around 300 surgeries and several years for clinicians to determine that Teflon was not suitable for acetabular cups. However, biomechanical data provided by a research engineer accurately predicted Teflon's poor wear performance by developing a wear tester and then identifying an alternative material with positive wear data within just three weeks [5].

The biomechanical bone models are prepared using bone substitutes or bone surrogates that include human cadaver bone, synthetic bone, and finite element models [5]. The natural bone is also used for dental implant biomechanical studies [6,7]. It has the advantage of simulating the in vivo environment, but it also has many limitations. It is costly as it takes a lot of effort and multiple steps are followed to prepare a sample. The storage and preservation of samples need chemical solutions which can affect the sample's tissue contents and hence its mechanical properties. When multiple samples are used, it is more likely that varied samples in terms of bone properties are formed and there is a chance of cross-infection. There are some ethical issues as well relating to the source [5,8].

In contrast to natural bone, synthetic bone models have high biomechanical fidelity, have low variability of specimens, are less costly, and are easy to use. These are available as sheets or blocks, and the composition can be adjusted for different bone qualities [8]. Recent generations of composite bone models with different percentages of filler contents show biomechanical properties like human bone under different loads [5]. The disadvantages related to synthetic models include no healing response and analyses can be limited to immediate postoperative behavior. These models lack soft tissue components, which otherwise apply controlled muscle loads [5]. At last, the synthetic models may behave similarly to human bones, but when tested as isolated specimens, local mechanical properties and mechanical responses to implants differ from human cadaver bones and can result in significantly different results between groups [5].

In multiple implant biomechanical studies, several materials have been used to represent bone such as epoxy resin [9,10], acrylic resin [11], and polyurethane (PU) resin [12]. Epoxy resin has been widely used in medical sciences as a substitute material for the simulation of many biological tissues like skin, muscle, bone, breasts, and lung tissue. One reason for its use is that its molding gives the advantage of wax-based materials but with varying mechanical properties. The adjustment in the quantity and type of filler particles added to unfilled resin gives us resin of varying densities and mechanical properties [13]. It is a thermoset resin. It has at least one or more epoxide groups in the molecule. The epoxide is also called oxirane. The oxirane functional group (a three-member ring formed between two carbon atoms and oxygen) is the main feature of the epoxy monomer. The 3D oxirane ring of epoxy is a cross-linked structure of cured epoxy [13]. Most of the commercially available epoxy resins are oligomers of diglycidyl ether of bisphenol A (DGEBA). When these oligomers react with the hardener, the epoxy resin gets cured and becomes a thermoset polymer [14].

PU foam is a group of lightweight porous materials with enormous interest due to their application in varying industries. PU skeleton is made up of two raw materials isocyanate and polyol which are derived from crude petroleum oil [15]. The polyols are building blocks, while isocyanates are the linking agents. Initially, when they were introduced, they were derived from polyester-based but nowadays polyether-based foams are produced. PU foam is vastly classified into flexible and rigid foams [16]. The rigid PU is comprised of a closed-cell structure [17]. In dental implant biomechanical studies, these rigid PU foams are widely used as a substitute for cadaveric bones because of their consistent, homogeneous structural properties and good mechanical properties. The American Society for Testing and Materials (ASTM) [18] has specified the use of PU foams as a material for biomechanical testing of bone implants.

Cadaveric bone, synthetic bone, and finite element models are all abstractions of reality, which can be used as tools to explain complicated behaviors with a biomechanical background. These models possess a unique set of advantages and disadvantages. It is important to keep them in mind when interpreting the findings [5]. Currently, there is no consensus as to whether synthetic bone, cadaveric bone, or finite element models provide results that best recapitulate the clinical experience, represent relevant outcomes, or have the most potential to influence standards of care. The three models are fundamentally different from one another, and it depends on the biomechanical feature to be measured as to the most appropriate to answer the respective research question [5].

The present study selected two mostly used synthetic materials as bone models. These materials were tested for their stress-distributing properties by strain gauge analysis. The strain was used as a measuring parameter. The null hypothesis was that there was no significant difference in the strain profile within the peri-implant region by using two different bone substitutes.

## Materials and methods

Study design 

It is an in vitro experimental biomechanical study investigating the suitability of two synthetic bone substitute materials, PU foam and epoxy resin, as models for peri-implant stress distribution analysis. Strain gauge analysis was employed to compare the stress distribution within the peri-implant region of each material. Two distinct implant designs were employed for each synthetic bone material to assess the reproducibility of the experimental results.

PU model 

**Figure 1:**
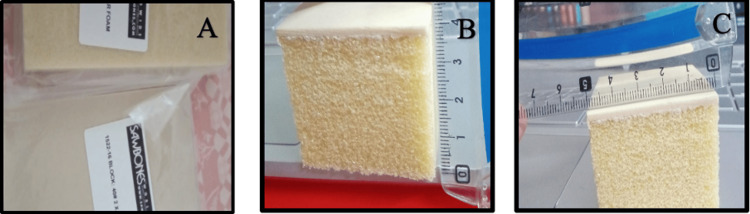
(A) PU foam sheets 40 and 15 PCF and (B, C) PU model blocks PU: polyurethane; PCF: pound per cubic foot

Rigid PU foam (Sawbones, Vashon, Washington, United States) was selected as a testing model substitute for bone [19]. Two different densities for PU foam were selected to design a D2 bone as per the Misch classification [2]. Misch has classified bone density into four types: D1 is dense cortical bone, D2 is porous cortical and coarse trabecular bone, D3 is porous cortical bone (thin) and fine trabecular bone, and D4 is fine trabecular bone. A D2 bone has dense to porous cortical bone and coarse trabecular bone [2]. In the present study, a 40 pound per cubic foot (PCF) (or lb/ft^3^) PU sheet was selected to mimic a dense cortical plate. It was 2 mm in thickness and was available in the dimensions of 13×18 cm. A 15 PCF porous PU was used to mimic the fine trabecular bone as shown in Figure 1. The two densities were bonded and cut into a cube shape to make a model of an area of 5 cm^3^ [20]. 

Epoxy resin model

**Figure 2:**
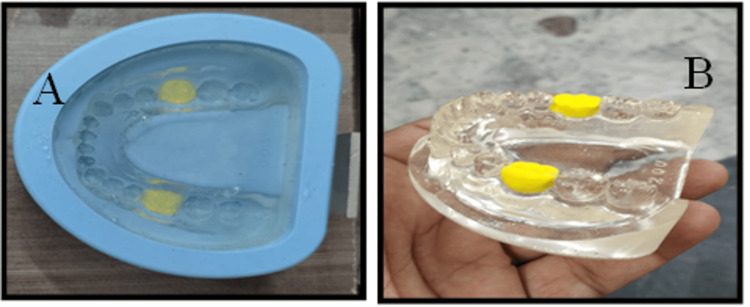
Epoxy resin model pour and final set

A self-curing epoxy resin (Polycraft ClearTop 35 Epoxy Water Clear Resin System, MB Fibreglass, Newtownabbey, Northern Ireland) was used. A standard epoxy resin has a density of 69.2 lb/ft^3^ or 1100 kg/m^3^ available in two liquid systems. The epoxy resin was mixed in a ratio of 1:1 and poured (Figure 2A) into a silicon mold (GreatLH, model number M8019, Chengdu, China) of a dentate mandibular arch. The first molars were blocked using modeling foam clay to create space for the implants to be placed in the model produced. All the bubbles were removed by tapping. The model was allowed to be cured for 24 hours (Figure 2B). 

Implant-supported prostheses were of two varying designs, design 1, a trapezoidal tapered thread-shaped implant (Tapered Pro, BioHorizons, Hoover, Alabama, United States), and design 2, a buttress thread-shaped implant (Grand Morse Neodent, Straumann, Basel, Switzerland), with diameters of 4 mm and 3.8 mm and lengths of 12 mm and 11.5 mm, respectively. Two implants per model (one implant (n=1) of each design) were drilled in both models using the manufacturer's instructions at a torque of 45-50 N/cm. 

Strain gauges (general-purpose BF 120 series, Beijing, China) were selected and placed in the coronal third region at 2 mm adjacent to the implant. The leads of the strain gauges were soldered to a Wheatstone bridge using a soldering gun. This setup was connected to an amplifier and data acquisition board. A data cable was used to transfer the data from the acquisition board to a computer software.

Loading of models

**Figure 3:**
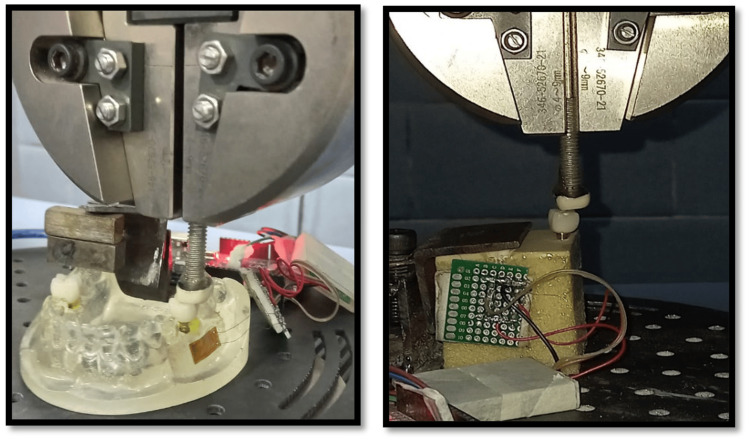
Epoxy resin model and PU foam model loading using a UTM PU: polyurethane; UTM: universal testing machine

The load application was done using a universal testing machine (UTM) (Shimadzu 50kN AGX Plus, Kyoto, Japan). A combined axial and non-axial load was applied through a porcelain fused to a metal prosthesis as an opposing tooth as shown in Figure 3. The load was applied up to 300 N and at the strain rate of 0.95 mm/sec. The corresponding strain values were recorded for 100 N, 150 N, 200 N, 250 N, and 300 N [9-10,12]. The readings were repeated thrice for each model. The data was analyzed using IBM SPSS Statistics for Windows, Version 22.0 (Released 2013; IBM Corp., Armonk, New York, United States). The comparison between groups was done using four-way ANOVA and post hoc Tukey's test. The p-value ≤0.05 was taken as statistically significant.

## Results

The strain values for PU foam and epoxy resin for four study groups at various loads are mentioned in Table 1.

**Table 1:** Strain values at different loadings for PU and epoxy resin models using four-way ANOVA test PU: polyurethane

Force (N)	PU foam			Epoxy resin			F-value
	Strain produced in design 1 (µε)	Strain produced in design 2 (µε)	P-value	Strain produced in design 1 (µε)	Strain produced in design 2 (µε)	P-value	
100	535±32.7	639±35.08	0.00	230±30.5	531±27.4	0.00	1712.502 (0.00)
200	841±48.1	1212±17.05		634±31.3	891±24.00		
300	1286±209	1812±22.5		906±22.3	1108±24.44		

**Figure 4:**
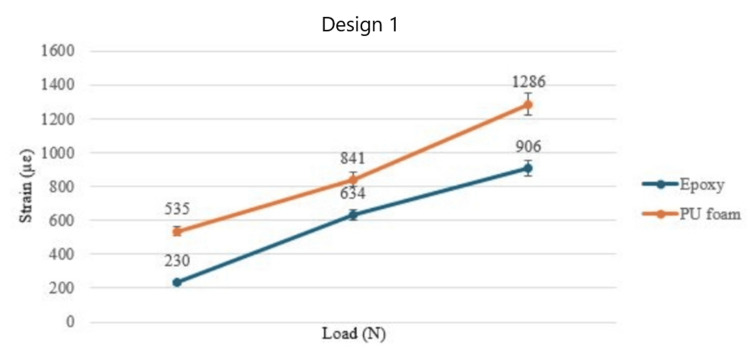
Graph showing the strain values for design 1 with two bone model materials under multiple loads PU: polyurethane

At mean biting force (MBF), the strain values for the PU foam model were 1286 µε and 1812 µε for designs 1 and 2, respectively, whereas the strain values from the epoxy model were 906 µε and 1108 µε for both designs. The PU foam models showed higher values for all the groups as compared to the epoxy models. However, both models showed similar trends in strain values. The difference between the strain values was statistically significant (p≤0.05). The strain values for PU foam were consistently higher for all four groups as shown in Figure 3 and Figure 4, respectively. 

**Figure 5:**
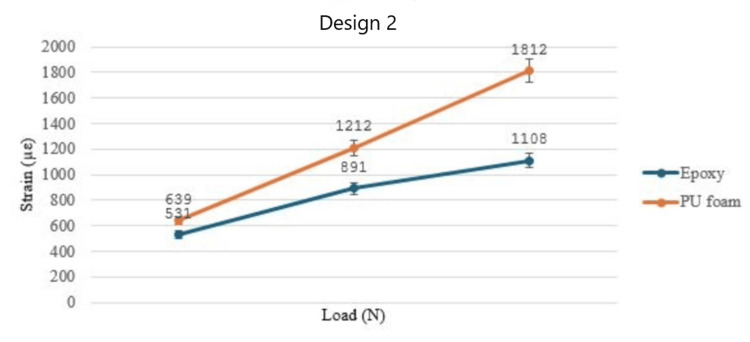
Graph showing the strain values for design 2 with two bone models materials under multiple loads PU: polyurethane

## Discussion

In this study, two types of bone substitutes were used for biomechanical testing: a PU foam and an epoxy resin. In the literature, many studies have used PU foam as a bone substitute [21-25]. However, many studies are still using traditional resin materials for the representation of bone [9-12]. PU is mentioned as a material of choice for the biomechanical testing of implants by the ASTM standard F1839-08 [18]. No study was found available in the literature comparing the two available substitute materials; hence, this study was designed to analyze both materials for use as an in vitro bone model.

The Frost theory states the strain values for physiological and pathological overloading. In the presence of a strain of ≤3000 µε, the net result is bone deposition. On the contrary, in the presence of strain ≥3000 µε, the net result is bone loss. This ultimately results in structural or biological failure appearing at the weakest parts of the system [2].

In this study, the strain values for both materials under different loading were compared using two different designs of implants. All the strain values for PU models were higher as compared to epoxy resin models with significant statistical differences (p≤0.05). The main reason for this significant difference was most likely due to the difference in the form of both materials. PU foam is similar not only in mechanical properties to the bone but also in its form. It has small pores ≤1 mm throughout its structure resembling trabecular bone. The presence of these pores decreases the overall surface area over which stress acts, and this in turn increases the amount of stress and thus strain produced per unit area of bone [1]. The values recorded are probably closer to actual values than those recorded using epoxy resin. However, with different strain values, the trend of increasing strain across different loadings was almost similar for both materials.

The method used for biomechanical study also may decide which model material to use. For example, if implant biomechanics are studied through photoelastic analysis, a birefringent resin material of any kind can be used; similarly, digital imaging correlation also makes use of resin material with the addition of speckles [26]. On the other hand, the biomechanical study using a strain gauge can make use of both kinds of synthetic materials (PU foam and resin). However, due to the morphological similarity of PU foam to trabecular bone, PU foam is preferred [12,25].

The mechanical properties of bone are crucial, that is, things like how strong it is, how it flexes, and how it responds to different kinds of forces. Researchers are constantly working to make synthetic bone models that match these properties more and more closely. This is vital for accurate testing and simulation. Synthetic composite bone models, such as those manufactured by Sawbones, offer a valuable alternative to biological bone for biomechanical testing, mitigating many of the limitations associated with using cadaveric or animal bone. These models, often composed of short glass fiber-filled epoxy resin, are designed to mimic the properties of cortical bone. Specifically, Sawbones' epoxy formulation, reinforced with short glass fibers, replicates key mechanical characteristics of cortical bone, including density, fracture toughness, strength, modulus, and hardness, achieving similarities to cadaveric cortical bone [27-29]. On the other hand, conducting research after implant design, i.e., using commercial implants, usually only focuses on exploring the expected advantages of available designs but rarely helps in resolving clinical issues [5].

A significant limitation of the present study is the small sample size. While the findings provide valuable insights into the stress distribution around dental implants, a larger sample size would enhance the statistical power of the study, allowing for more definitive conclusions and the identification of subtle differences in stress distribution patterns. Future research with a more extensive and diverse sample pool is necessary to validate these findings and further explore the implications for clinical practice. Biomechanical studies are an important but neglected component of dentistry. Successful biomechanical research requires a strong partnership between surgeons and scientists.

## Conclusions

The comparative analysis of PU foam and epoxy resin as bone substitute materials for biomechanical testing revealed that PU foam demonstrated higher strain values, attributed to its porous structure that better mimics trabecular bone. These findings suggest PU foam may provide strain measurements closer to physiological values. However, the choice of material should align with the specific method and goals of each biomechanical study.
